# Evaluation of polarization rotation in the scattering responses from individual semiconducting oxide nanorods

**DOI:** 10.1063/1.4948267

**Published:** 2016-04-22

**Authors:** Daniel S. Choi, Manpreet Singh, Hebing Zhou, Marissa Milchak, Brian Monahan, Jong-in Hahm

**Affiliations:** Department of Chemistry, Georgetown University, 37^th^ & O Sts. NW., Washington, DC 20057, USA

## Abstract

We investigate the interaction of visible light with the solid matters of semiconducting oxide nanorods (NRs) of zinc oxide (ZnO), indium tin oxide (ITO), and zinc tin oxide (ZTO) at the single nanomaterial level. We subsequently identify an intriguing, material-dependent phenomenon of optical rotation in the electric field oscillation direction of the scattered light by systematically controlling the wavelength and polarization direction of the incident light, the NR tilt angle, and the analyzer angle. This polarization rotation effect in the scattered light is repeatedly observed from the chemically pure and highly crystalline ZnO NRs, but absent on the chemically doped NR variants of ITO and ZTO under all measurement circumstances. We further elucidate that the phenomenon of polarization rotation detected from single ZnO NRs is affected by the NR tilt angle, while the phenomenon itself occurs irrespective of the wavelength and incident polarization direction of the visible light. Combined with the widespread optical and optoelectronic use of the semiconducting oxide nanomaterials, these efforts may provide much warranted fundamental bases to tailor material-specific, single nanomaterial-driven, optically modulating functionalities which, in turn, can be beneficial for the realization of high-performance integrated photonic circuits and miniaturized bio-optical sensing devices.

Effective manipulation of optical and electro-optical signal is critical to the successful operations of optical modulators, switches, interconnects, and photodetectors in integrated photonic circuits and optoelectronic devices.^1–6^ In this regard, optical anisotropy of semiconductor materials has drawn considerable research interest, particularly focusing on the manipulation of polarization rotation and phase retardation of linearly polarized light.^4–6^ As a part of these efforts, important aspects such as device size reduction and working wavelength range are actively pursued for further improvements in advanced optical and photonic devices. Recent progresses to precisely tailor the geometry and composition of materials at nanometer length scales have continued to push miniaturization of optical components,^7–9^ down to the ultra-compact regime of exploiting individual nanomaterials as functional optical elements instead of their ensembles. However, only limited numbers of experimental work are available in the literature attempting to provide insight into the precise nature of light interaction with individual nanomaterials.^10–15^ Yet, such an understanding is imperative to tune single nanomaterial devices with desired optical functionality. As for the working wavelength range, many previous research efforts have successfully demonstrated manipulations of infrared (IR) light and operations at telecommunication wavelength ranges.^1–6^ However, parallel efforts are still highly warranted for the development of visible and ultraviolet light counterparts, whose modulation can be particularly useful in bio-optical imaging and sensing.^16–19^

In this Letter, we evaluate the light-matter interactions of technologically important semiconducting oxide nanorods (NRs) at the single nanomaterial level, while controlling key light-matter interaction parameters such as the wavelength and polarization direction of light, the NR tilt angle, and the analyzer angle. Pure and chemically doped NRs with comparable physical dimensions are systematically characterized for their material-dependent elastic scattering responses upon interactions with visible light. Specifically, we have chosen single NRs of ZnO as well as indium tin oxide (ITO) and zinc tin oxide (ZTO) due to their well-known functional benefits as lasers,^20,21^ light emitters,^22–25^ waveguides,^26–30^ photovoltaics,^31^ and biosensors.^32,33^ We subsequently elucidate an intriguing scattering response of rotation in the polarization direction of the scattered light from individual NRs. We further identify that polarization rotation of the scattered light is material-dependent, seen only from the pure structures of ZnO NRs but not from the doped materials of ITO and ZTO NRs. We also ascertain that the polarization rotation effect of the ZnO NR-scattered light is influenced by the NR tilt angle, and the phenomenon occurs irrespective of the wavelength and incident polarization direction of the visible light. Our efforts provide much needed insight into the material-dependent, visible light-matter interaction behaviors of individual semiconducting oxide nanomaterials and further offer fundamental bases to modulate scattered light in single ZnO NR devices by exploiting different material types and effective angles of the light-matter interaction.

Individual NR samples of ZnO, ITO and ZTO were synthesized by using a home-built chemical vapor deposition (CVD) reactor as reported earlier.^24,34–37^ The sizes, morphologies, and crystal structures of the as-grown nanomaterials were also reported previously along with their scanning electron microscopy (SEM) and X-ray diffraction (XRD) data.^24,34–37^ The typical diameters and lengths of the NRs employed in our measurements range from 150 to 300 nm and from 5 μm to 30 μm, respectively. A home-built series of optical components were configured in a dark-field (DF) mode on a commercially available optical microscope of Olympus BX51F in order to optimally collect the inherently weak and hard-to-detect scattering signals from individual nanomaterials. The measurement arrangement involved two scattering geometries, one with a forward DF scattering (FDF) and the other with a reflected DF scattering (RDF) configuration. In the FDF measurements shown in Figure 1(a), a linearly polarized diode laser with either λ = 488 nm or 642 nm (Spectra Physics Excelsior-PS-DD-CDRH) entered the NR plane via an oil-immersion DF condenser (Numerical Aperture, NA = 1.2 ∼ 1.4) from below the sample stage after passing through a half-lambda (HL) wave plate. The HL plate controlled the incident direction of the linearly polarized laser whose oscillation is referred to as **E**_∥_ (and **E**_⊥_ when the polarization direction is within (and perpendicular to) the plane of incidence, as illustrated in Figure 1(b). Figure 1(b) also displays the NR tilt angle of θ formed by the NR main axis and the y-axis, where the y-axis refers to the intersecting axis between the plane of incidence (y-z) and the sample plane (x-y). Scattering signals from individual NRs were collected with a 40x plan apochromatic objective lens (Olympus PlanSApo, NA = 0.90). An analyzer was placed between the microscope tube lens and the charge-coupled-device detector (QImaging Exi Blue CCD camera, Surrey, Canada). The RDF mode involved wide-field, unpolarized illumination (a 100 W, 12 V halogen lamp) above the sample plane and subsequent collection of the scattering signal in the backward direction towards a DF objective lens. The RDF signals were subsequently measured with 20x (Olympus MPlanFL N, NA = 0.45) and 50x (Olympus MPlanFL N, NA = 0.80) DF objective lenses. The NR samples were prepared for the FDF and RDF investigation using a refractive index-matching assembly method described elsewhere.^13,14^ We have measured and analyzed the elastic scattering responses from a large number of ZnO, ITO and ZTO NRs, over 100 NRs in total. Herein, we report the material-governed, optical phenomenon pertaining to the presence and absence of the polarization rotation in the NR scattered light. The elastic scattering data reported herein are collected at the same wavelength as that of the incident light and, hence, they are not from bandgap photoluminescence or inelastic Raman scattering.

Representative scattering data obtained from individual ZnO NRs are shown in Figures 2 and 3, specifically by presenting the FDF and RDF scattering characteristics measured from a 10.15 μm-long ZnO NR with the tilt angle of 70^o^. The 3-dimensional (3D) contour plots simultaneously chart the scattering intensity, the position along the NR long axis, and the analyzer angle under the different incident light settings controlled by the wavelength and the polarization direction of the incoming laser. As reported earlier, the scattering response from individual NRs can either be continuous or discontinuous, depending on the NR tilt angle.^15^ With the θ of 70^o^, the FDF scattering patterns from the ZnO NR are expected to be discontinuous, as shown in Figure 2, due to the dominant component of **E**_⊥_ projected onto the sample plane, mimicking the highly localized scattering signal discretely monitored only at the two NR ends at high θ angles approaching 90^o^.^15^

Furthermore, careful examination of the ZnO NR scattering profiles in the 3D contour plots in Figure 2 clearly reveal additional asymmetry that has never been elucidated before. Asymmetric scattering characteristics are identified not only for the scattering intensity at different positions along the NR length but also in terms of the analyzer angles corresponding to the maximum and minimum scattering intensity at each NR end. The novel observation implies that the scattered light from the ZnO NR goes through a polarization rotation effect, and the electric field oscillation changes from one to the other end of the ZnO NR, as schematically illustrated in the right panel in Figure 1(c). This rotation of ZnO NR-scattered light occurs irrespective of the wavelength and the polarization direction of the incident light, as evidenced by the data presented in Figure 2(a)-2(d). However, we note that the rotation effect of the polarized scattered light seen from individual ZnO NR scattering is influenced by the NR tilt angle. We observe no measurable rotation of the polarized scattered light from the ZnO NRs of θ = 0^o^, regardless of the incident polarization. In general, we also observe that the angle shift in the polarization oscillation of the scattered light becomes larger as the NR tilt angle of θ increases. And the polarization rotation effect in the scattered light occurs more pronouncedly on the ZnO NRs of the intermittent and discrete scattering nature^15^ relative to the continuous type.

A series of images in Figure 2(e) substantiates the polarization rotation effect in the scattered light monitored from the ZnO NR. The FDF scattering snapshots for the θ = 70^o^ ZnO NR unambiguously show the switching of the bright ends at different analyzer angles. For example, for the incident light setting of 488 nm and **E**_∥_, the left end of the ZnO NR scatters the strongest when the analyzer rotation is parallel to **E**_∥_. When the analyzer angle gradually increases, the left end of the NR scatters more weakly, while the scattering from the right NR end becomes stronger. When the analyzer angle is perpendicular to **E**_∥_, scattering from the left NR end significantly diminishes and that of the right end becomes intense instead. Similar trends are identified in all FDF scattering snapshots shown in Figure 2(e). As a comparison, the RDF scattering image of the same ZnO NR is shown in the last row of Figure 2(e). As this RDF measurement is carried out with wide-field illumination of unpolarized light, scattering intensities along the NR long axis show a continuous pattern, unlike the discontinuous FDF scattering configurations.

The set of 2D surface plots in Figure 3 is the FDF scattering responses from the ZnO NR, clearly revealing the polarization rotation effect of the scattered light. The data were gathered at different analyzer angles for each incident light setting specified. The extent of optical rotation can be estimated by the analyzer angles corresponding to the scattering maximum/minimum at each end of the ZnO NR. As a guide to the eye, those analyzer rotations are marked with white and black arrows to indicate the scattering maximum and minimum, respectively. The degree to which the analyzer angle yielding the scattering maximum (or minimum) shifts from one to the other end of the ZnO NR is close to 90^o^ for λ = 488 nm while that for the case of λ = 642 nm is approximately 10-25^o^. Therefore, our data indicate that the phase shift in the ZnO NR-triggered polarization rotation is expected to be higher for a light-matter interaction probed with a shorter wavelength of light. The observed rotation of the polarization from one end to the other end of the ZnO NR may be related to the doubly refracting nature of ZnO. The high birefringency of ZnO exhibits a difference of approximately 0.018 between the ordinary and the extraordinary refractive indices at the visible wavelength range.^38,39^ The ZnO NRs used in our experiments are grown exhibiting high crystallinity and chemical purity which was evidenced by the diffraction and photoluminescence spectra of the pristine crystals.^18,19,40^ Therefore, individual ZnO NR crystals may produce a birefringence-originated phase difference which is known to be inversely proportional to the wavelength of the light.^16,41^

We further examined other related nanomaterials for their scattering behaviors at the individual NR level and assessed whether a comparable polarization rotation effect is also present in doped variants of semiconducting oxide NRs. The typical FDF scattering plots from a single ITO and ZTO NR with comparable physical dimensions and light-matter interaction angles to those of the previously discussed ZnO NR cases are displayed in Figure 4(a) and 4(b), respectively. In all circumstances of the visible wavelength and incident polarization, the resulting scattering patterns at each end of the ITO and ZTO NRs were symmetric in terms of the analyzer angles enabling the passing of the maximum/minimum scattering signals. Consequently, no rotation in the polarization direction of the scattered light occurred in the scattering from ITO and ZTO NRs. Hence, we confirm that the intriguing optical rotation, persistently observed from ZnO NRs, is absent in scattered signals from ITO and ZTO NRs regardless of the incident light setting. The lack of the polarization rotation phenomenon in the scattered light from these doped NRs suggests that the birefringent nature of the pure materials, through their well-ordered atomic arrangements in the crystals, is lost due to the introduction of the doping elements. Chemical dopants of In and Sn in ITO and ZTO NRs, respectively, lead to random distributions of the dopant elements in the otherwise pure and crystalline NRs of SnO_2_ and ZnO which, in turn, can serve as chemical and structural defect sites. Such chemical and structural perturbations may explain the absence of any phase shift in the scattered responses of the ITO and ZTO NRs seen in our experiments. Compared to the presence of optical rotation in ZnO NRs, no change in the oscillation directions of the scattered electric fields from ITO and ZTO NRs is observed, as depicted in the left panel in Figure 1(c). Further work is underway in order to determine the exact origin of the polarization rotation of the scattered light that was persistently observed from individual ZnO NRs but absent on ITO and ZTO NRs.

Our findings may serve as a new guidance to tailor material-specific, single nanomaterial-driven, optically modulating functionalities in photonic and sensing devices. Our endeavors may be particularly valuable in developing biomedical sensors based on individual ZnO NRs by equipping them with additional modalities of polarization rotation and phase shift. In addition, with the demonstrated application of ZnO NRs as nanolasers and subwavelength waveguides,^21,27,28,30^ understanding the factors influencing their polarization rotation effect can be highly useful in building single ZnO NR-based, optically functional elements.

In summary, we have examined the light-matter interactions between visible light and the NRs of ZnO, ITO, and ZTO at the single nanomaterial level, while systematically controlling the key parameters such as the wavelength and polarization direction of light, the NR tilt angle, and the analyzer angle. We subsequently report the intriguing optical phenomenon of polarization rotation in the scattered light, which is confirmed to be present on the chemically pure and highly crystalline ZnO NRs, but absent on the chemically doped NR variants of ITO and ZTO with comparable physical dimensions and light-matter interaction geometries to ZnO. We further elucidate that the material-dependent polarization rotation of the scattered light is affected by the NR tilt angle, although the phenomenon itself occurs irrespective of the wavelength and incident polarization direction of the visible light for ZnO NRs.

## Figures and Tables

**FIG. 1.**
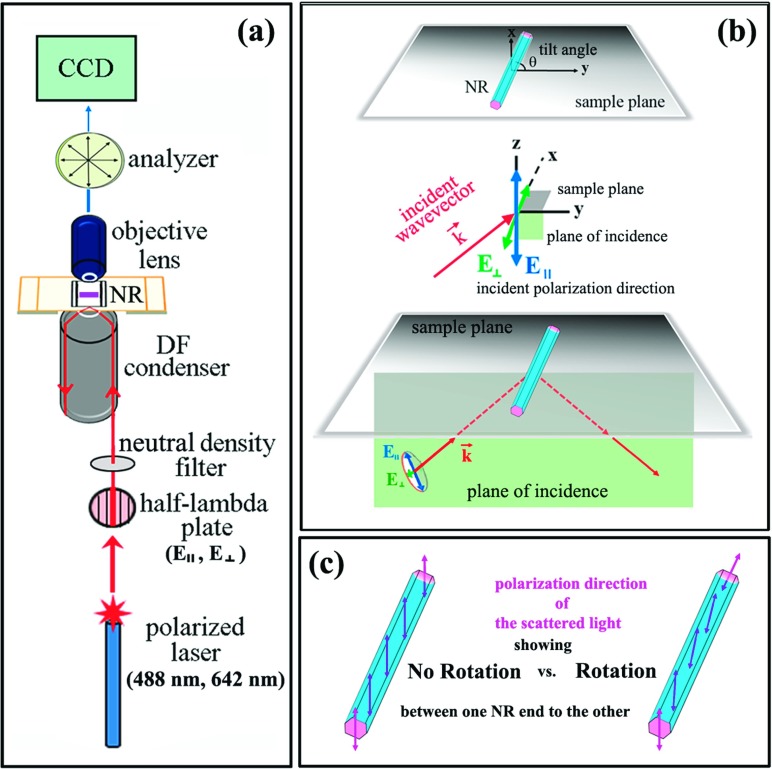
Schematic representations of the FDF scattering measurement setup to examine the light-matter interaction profiles from ZnO, ITO, and ZTO NRs. **(a)** The overall instrumental configurations are shown. Linearly polarized lasers of the two different incident wavelengths (λ = 488 nm or 642 nm) are employed to examine the NR scattering behaviors. **(b)** Key measurement geometries of the NR tilt angle (θ), the incident polarization direction (**E**_∥_ or **E**_⊥_), and the incident light wavevector are shown along with the sample plane (x-y) and the plane of incidence (y-z). **(c)** The light-matter interaction cases exhibiting no rotation and a rotation in the polarization direction (marked with double headed arrows) of the scattered light are depicted in the left and right NR, respectively.

**FIG. 2.**
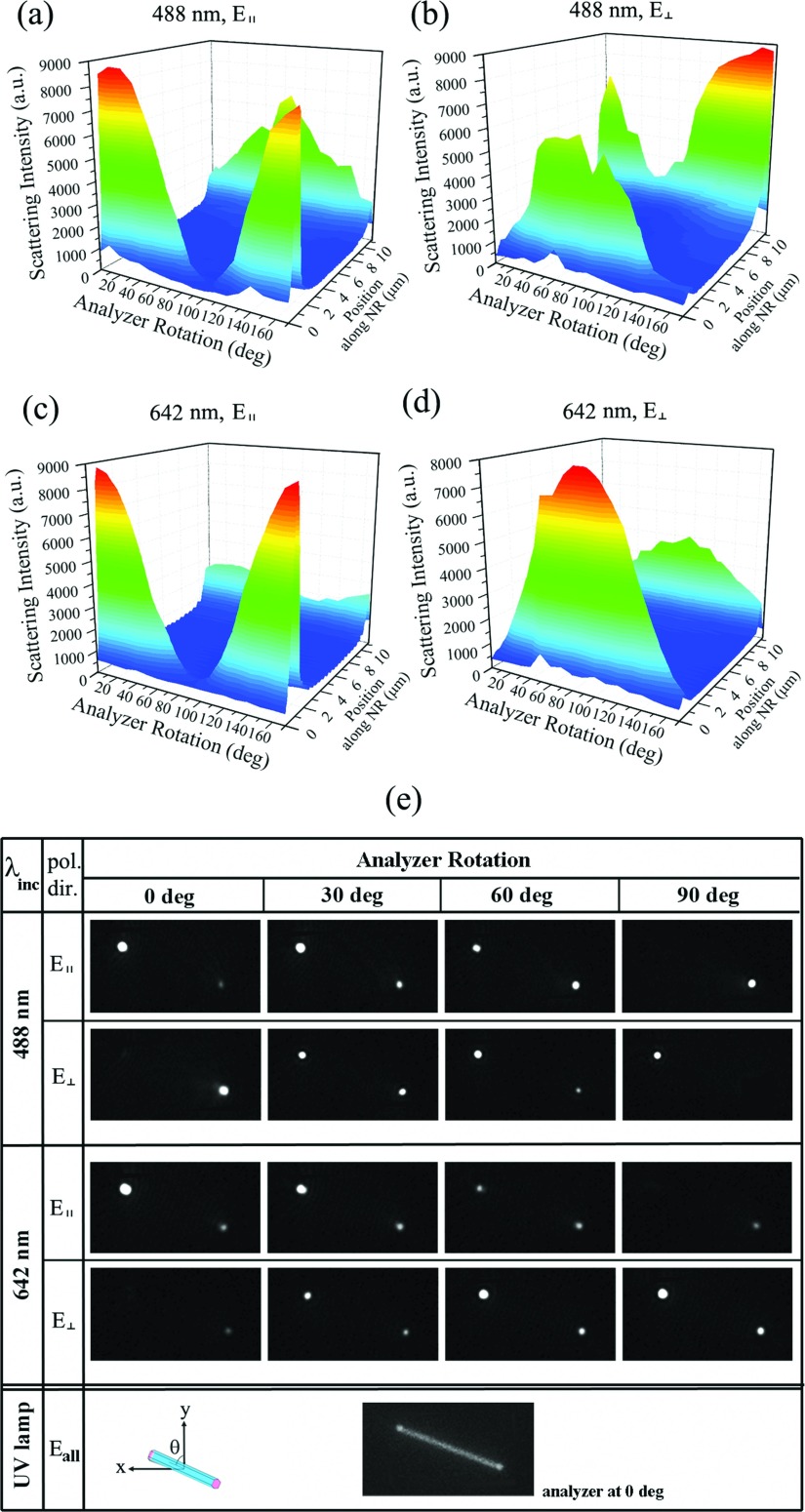
FDF and RDF scattering signals from a 10.15 μm-long ZnO NR oriented at θ = 70^o^ are profiled. **(a-d)** The 3D contour plots show the representative NR scattering responses characterized by using different combinations of incident wavelengths and polarization directions which are (a) 488 nm, **E**_∥_, (b) 488 nm, **E**_⊥_, (c) 642 nm, **E**_∥_, and (d) 642 nm, **E**_⊥_. The scattering intensity for each case is graphed as a function of the analyzer angle and the position along the ZnO NR. **(e)** The snapshot series of the FDF patterns display the typical scattering images taken from the ZnO NR for the specified experimental parameters of λ, **E**, and the analyzer angle. In addition, the RDF scattering image taken with the wide-field illumination of unpolarized light is provided in the last row.

**FIG. 3.**
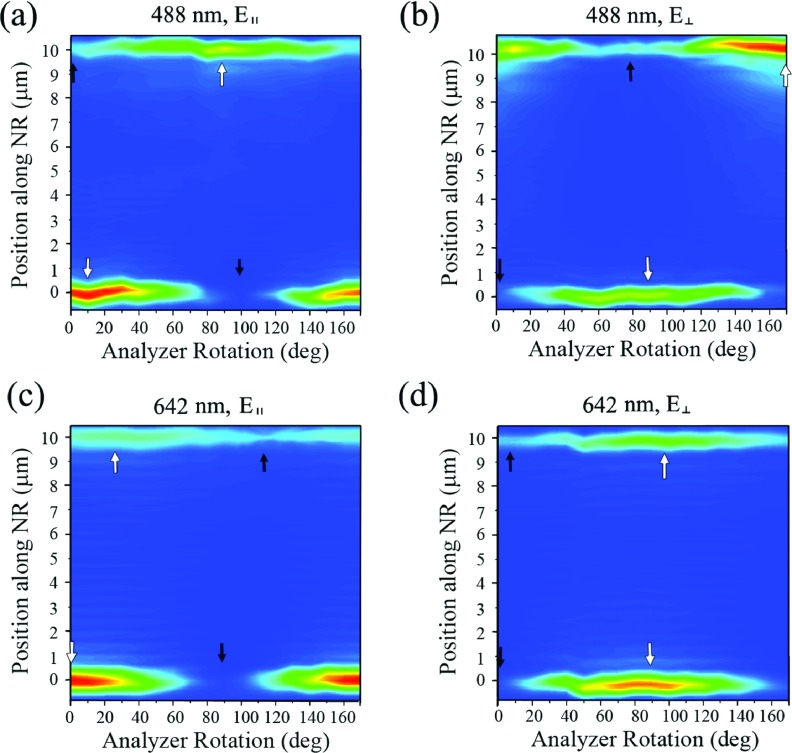
The 2D surface plots of the FDF scattering from the ZnO NR (10.15 μm-long with θ of 70^o^) clearly display the optical rotation phenomenon in the polarization of the scattered light revealed by varying the analyzer angle, whose oscillation rotates from one end to the other end of the ZnO NR. The incident light setting used for each measurement is **(a)** 488 nm, **E**_∥_, **(b)** 488 nm, **E**_⊥_, **(c)** 642 nm, **E**_∥_, and **(d)** 642 nm, **E**_⊥_. As a guide to the eye, the scattering maximum (and minimum) angle on each end of the NR is indicated with white (and black) arrows.

**FIG. 4.**
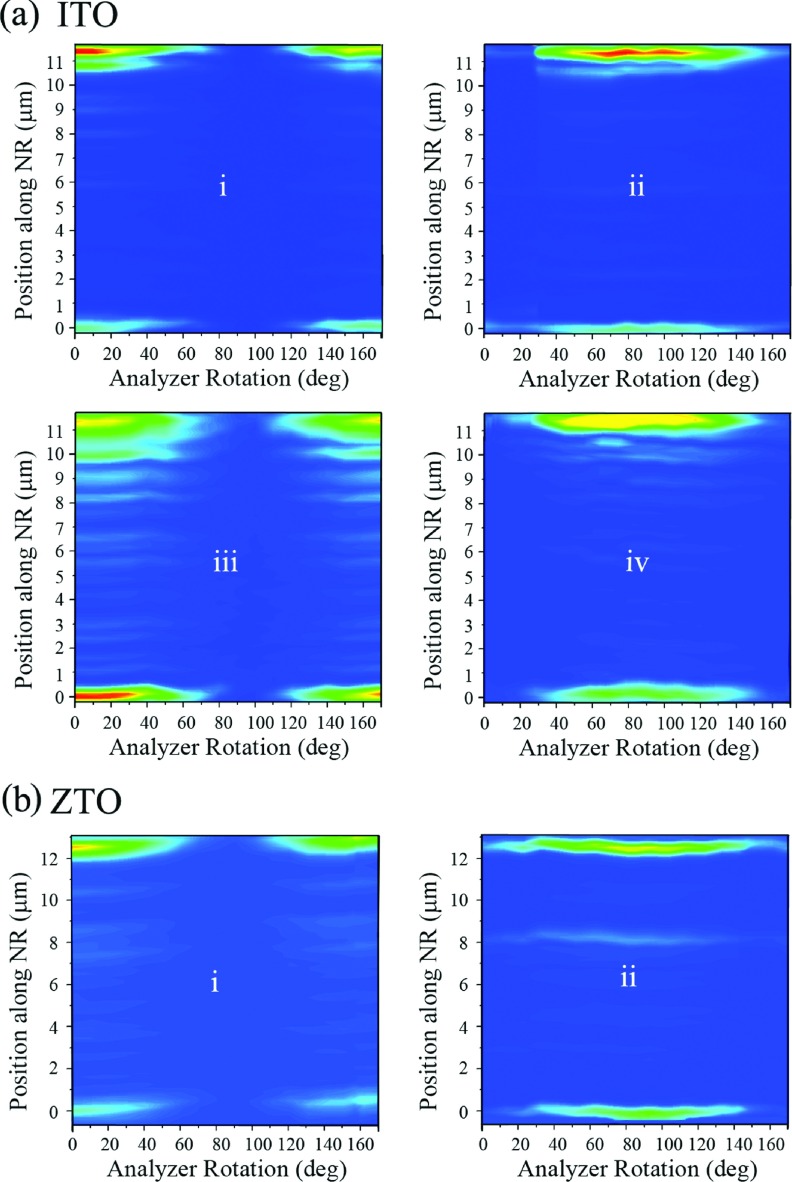
Typical FDF scattering profiles of ITO and ZTO NRs of similar tilt angles as the ZnO NR shown previously are presented. In all cases, no rotation in the polarization direction of the scattered light is observed. **(a)**The scattering intensities from an individual ITO NR (11.3 μm in length, θ = 70^o^) are graphed as a function of the analyzer angle and the position along the NR in the 3D contour plots for the incident light of **(i)** 488 nm, **E**_∥_, **(ii)** 488 nm, **E**_⊥_, **(iii)** 642 nm, **E**_∥_, and **(iv)** 642 nm, **E**_⊥_. **(b)** The scattering intensities from a ZTO NR (12.41 μm in length, θ = 75^o^) are displayed as a function of the analyzer angle and the position along the NR under the incident light of **(i)** 642 nm, **E**_∥_, and **(ii)** 642 nm, **E**_⊥_.
